# Draft Genome Sequence of a Bacterium Isolated from Hypersaline Soil in Sonora, Mexico: *Halomonas* sp. Strain BLLS135

**DOI:** 10.1128/mra.01409-20

**Published:** 2022-02-17

**Authors:** Dora Alicia Rodríguez-Franco, Sergio de los Santos-Villalobos, Juan Carlos Coronado-Corral, María Isabel Estrada-Alvarado, Luis Alberto Cira-Chávez

**Affiliations:** a Departamento de Biotecnología y Ciencias Alimentarias, Instituto Tecnológico de Sonora, Ciudad Obregón, Sonora, Mexico; b Departamento de Ciencias Agronómicas y Veterinaria, Instituto Tecnológico de Sonora, Ciudad Obregón Sonora, Mexico; University of Southern California

## Abstract

*Halomonas* sp. strain BLLS135 was isolated from hypersaline soil in Mexico. Here, we present the draft genome of this strain. Its genome has 2,861 protein-coding genes, 63 tRNAs, two 16S rRNAs, five 5S rRNAs, and a single copy of 23S rRNA, with a GC content of 63.5%.

## ANNOUNCEMENT

*Halomonas* is an aerobic and halophilic bacterial genus that belongs to the *Gammaproteobacteria* class (1, 2). This genus grows under low and high salinity and on various carbon sources (3, 4) and even utilizes ectoine as the sole carbon and nitrogen source (5). In addition, strains of this genus have shown the ability to produce hydrolytic enzymes (6–8). For this reason, our research group studied the hydrolytic capacity of strain BLLS135, which showed great metabolic diversity associated with the production of this type of enzyme and the response to different types of stress. Thus, it was decided to sequence the genome to explore new biotechnological capabilities.

Strain BLLS135 was isolated from hypersaline and alkaline soil on the coast of Sonora, Mexico (27°17′20″'N, 110°25′22″W). The soil samples were collected according to NOM-021-RECNAT-2000 (9). The isolation of bacteria was carried out by serial dilutions. All isolates were purified by sequential streaking on solid marine agar (BD Difco). The isolates were preserved in a microbial culture collection named Colección de Microorganismos Edáficos y Endófitos Nativos (www.itson.mx/COLMENA) (10).

**FIG 1:**
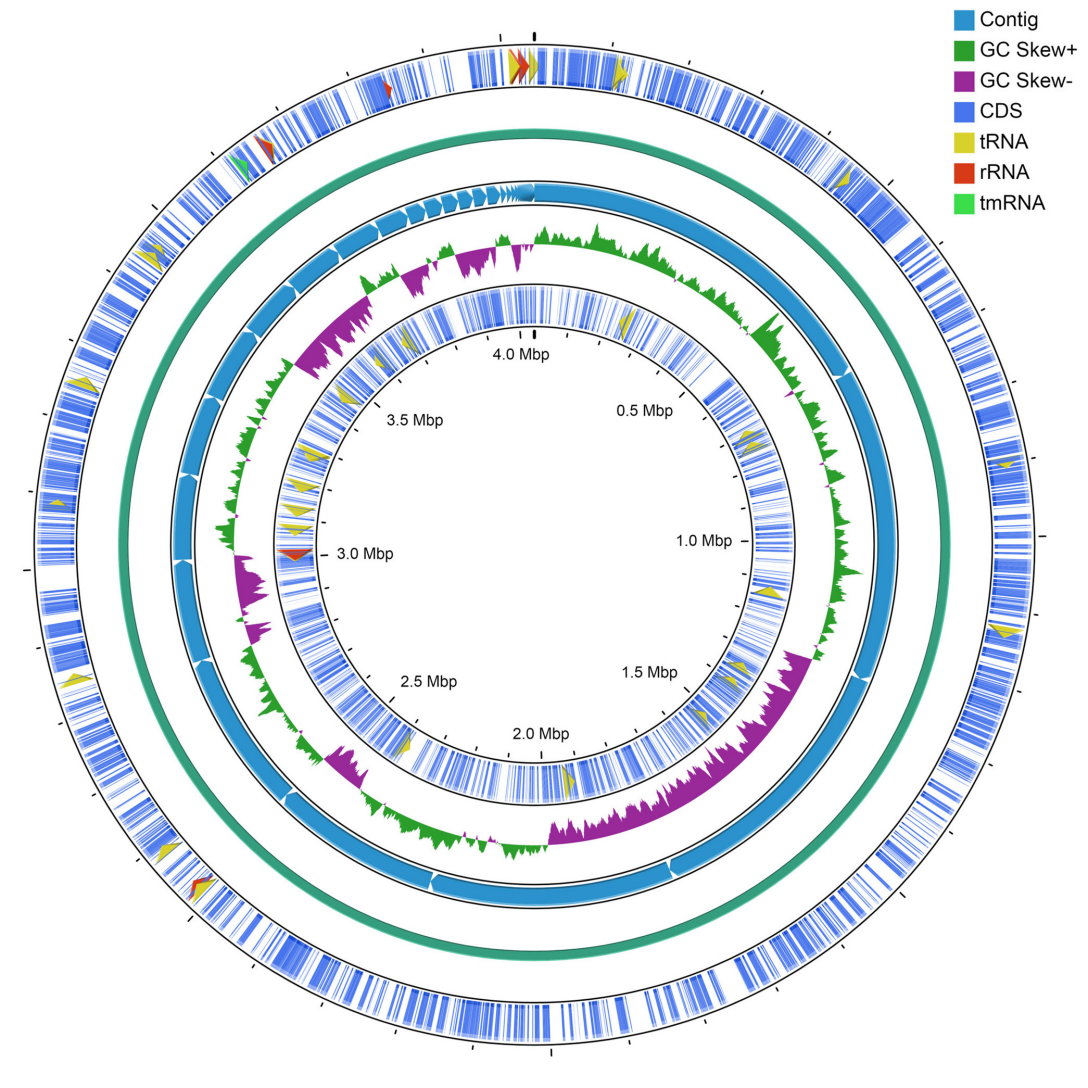
Circular chromosome map of *Halomonas* sp. strain BLLS135, showing the distribution of CDSs, tRNAs, rRNAs, and transfer-messenger RNAs (tmRNAs) and the GC content skew (50% of the total base pair window). The solid blue-green ring is the backbone of the sequence. The map was generated using the CGView Server beta online tool.

The genomic DNA was extracted from strain BLLS135 after growth in marine broth (BD Difco), incubation at 37°C for 20 h, and purification according to the phenol-chloroform method (11). High-quality DNA (optical density at 260 nm [OD_260_]/OD_280_ of 1.8 to 2.0) (total amount of DNA, ≥2 μg; concentration, ≥50 ng/μL) was sequenced by using the Illumina MiSeq platform (2 × 300 bp; Illumina, USA). Next-generation sequencing (NGS) library preparation was carried out by using the TruSeq DNA Nano kit for Illumina platforms, according to the manufacturer’s instructions. The sequencing process generated a total of 920,159 raw reads, with 114× coverage. The quality of the raw reads was evaluated using FastQC v0.11.7 (12), and Trimmomatic v0.32 (13) was used to remove adapter sequences and low-quality bases (minimum quality score of 33). Thus, we obtained 838,822 high-quality reads (91.16%). *De novo* assembly of the trimmed reads was carried out by SPAdes v3.10.1 (14), using the parameter --careful for error correction in reads and –cov-cutoff auto (SPAdes automatically computes the coverage threshold by using a conservative strategy). The draft genome consisted of 45 contigs of >500 bp (minimum size, 526 bp; maximum size, 688,674 bp; *N*_50_, 446,086 bp; *L*_50_, 4). The final assembly contained 4,042,999 bp, with a coverage depth of 114× and a GC content of 63.5%. These contigs were ordered by Mauve v2.4.0 (15, 16) using the type strain Halomonas elongata DSM 2581 (GenBank accession number GCA_000196875.2) as a reference genome, based on the greatest 16S rRNA gene similarity (>99%) according to the EzBioCloud 16S rRNA-based identification tool (16). The circular chromosome map was generated using the CGView Server (17) (Fig. 1), which provided DNA coding sequences (CDSs), tRNAs, RNAs, and GC skew. Default parameters were used for all software.

Genome annotation was performed by the NCBI Prokaryotic Genome Annotation Pipeline (PGAP) (18–20). This draft genome is predicted to contain 3,757 CDSs, 3,753 protein-coding genes, 76 RNA genes (62 of which are tRNAs, 10 of which are rRNAs, and 4 of which are noncoding RNAs [ncRNAs]), and 4 pseudogenes, with a mean GC content of 63.5%. Putative functions could be assigned to 2,861 protein-coding genes. Genome analysis revealed genes related to hydrolytic enzyme production, including β-*N*-acetyl-glucosaminidase, β-lactamase, α-glucosidase, β-xylosidase, and lysozyme, among others. This genomic information provides the first insight into the genetic background of *Halomonas* sp. strain BLLS135.

### Data availability.

This draft genome sequence was deposited in DDBJ/ENA/GenBank under the BioProject accession number PRJNA669612 and the BioSample accession number SAMN16456195. The raw reads were deposited in the Sequence Read Archive (SRA) under accession number SRX9621010. The genomic version described in this paper is version JADDHN000000000.1. The contigs are available under the accession numbers JADDHN010000001 to JADDHN010000045.
